# Role of C-reactive protein in disease progression, diagnosis and management

**DOI:** 10.15190/d.2023.18

**Published:** 2023-12-31

**Authors:** Sarah Ali, Aiza Zehra, Muhammad Umair Khalid, Momina Hassan, Syed Imran Ali Shah

**Affiliations:** ^1^MBBS, CMH Lahore Medical University, Lahore, Pakistan; ^2^Head of Department, Department of Biochemistry, CMH Lahore Medical University, Lahore, Pakistan

**Keywords:** C-Reactive Protein, CRP, Inflammation, Biomarker, Acute phase protein.

## Abstract

C-reactive protein (CRP) is a ring-shaped pentameric protein synthesized in the liver via CRP gene transcription. It is an inflammatory marker, whose serum levels can be measured using traditional and high-sensitivity tests. In healthy adults, the normal CRP serum concentrations vary between 0.8 mg/L and 3.0 mg/L. These can be grouped into low-, moderate-, and high-risk categories according to CRP levels of less than 1, 1-3, and greater than 3 mg/L, respectively. Elevated levels have been observed in infections, autoimmune diseases, neurodegenerative disorders, and malignancies. However, it is not specific to any disease. Serum CRP levels have also been shown to indicate the risk of cardiovascular disease, owing to their role as inflammatory markers in atherosclerosis, coronary artery disease, and peripheral arterial disease. Furthermore, its role in autoimmune diseases, such as Systemic Lupus Erythematosus and rheumatoid arthritis, and its involvement in the development of cancers, including breast, colorectal, ovarian, prostate, and lung cancers, have also been studied. The involvement of CRP in determining the course of infection and differentiating between bacterial and viral infections has also been investigated. This review summarizes the published literature on C-reactive protein and its role in disease management and progression.

## SUMMARY

1. Introduction
2. CRP in Disease Progression
  - 2.1 CRP in cardiovascular disorders
  - 2.2 CRP and Autoimmunity
  - 2.3 CRP in Sepsis and Infection
  - 2.4 CRP and Cancer
3. CRP in Diagnosis
  - 3.1 CRP in Infection and Sepsis
  - 3.2 CRP in Osteomyelitis
  - 3.3 CRP in Inflammatory Bowel Disease
4. CRP in Disease Management
5. Limitations and Future discussions
6. Conclusion

## 1. Introduction

C-reactive protein (CRP) is an acute-phase reactant that can increase 1000-fold in systemic infections, trauma, and malignancies. CRP is mainly synthesized in the liver and is a vital component of the innate immune system. CRP is important in host defense and the clearance of apoptotic cells by binding to foreign pathogens and damaged cells. In 1930, CRP was first described by Tillet and Francis and found to be elevated in patients with pneumococcal pneumonia^1^. The use of CRP as a diagnostic tool and marker of disease progression and treatment response has been increasingly recognized in recent years. This narrative review aimed to summarize the current state of knowledge regarding the implications of CRP in disease progression, diagnosis, and management. A comprehensive literature search was conducted using various databases including PubMed, Google Scholar, Lens, and Dimensions. The search was conducted using the following keywords: "C-reactive protein," “CRP” with "inflammation," "disease progression," "diagnosis," and "management."

**Figure 1:**
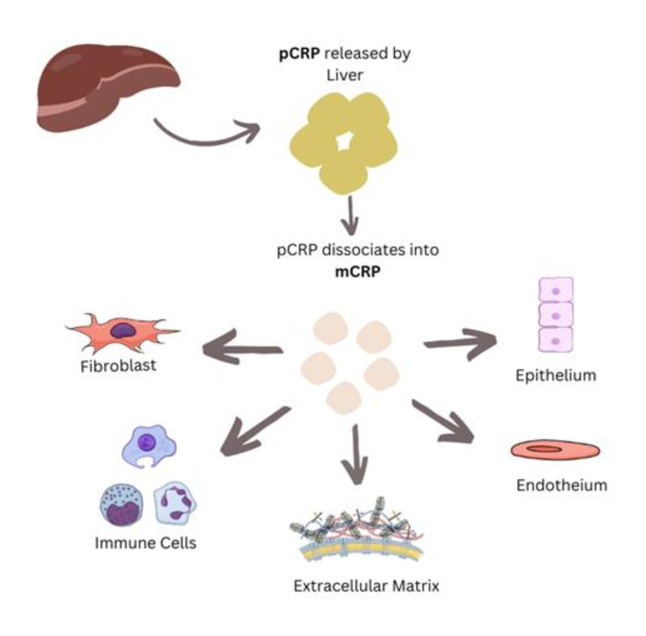
Schematic representation of pCRP and mCRP interactions. pCRP is released from hepatocytes due to inflammation and circulates through the systemic vasculature. pCRP once dissociated into mCRP becomes highly active. mCRP in turn interacts at different sites of inflammation including Immune Cells, epithelial cells, endothelial cells, fibroblasts, and part of the extracellular matrix.

CRP mainly belongs to the pentraxin family of calcium-dependent ligand-binding plasma proteins, mainly found as pentamers in circulation, also known as native CRP (nCRP)^2^. The pentameric form is primarily synthesized in hepatocytes but can also be synthesized in smooth muscle cells, macrophages, lymphocytes, and adipocytes^3^. It also exists in its monomeric form (mCRP) in plasma^4, 5^. In contrast to the pentameric form, which exerts both pro-inflammatory and anti-inflammatory actions, the monomeric form exerts a pro-inflammatory response in endothelial cells, endothelial progenitor cells, leukocytes, and platelets, and may amplify the inflammatory response^5^ (Figure 1). Its synthesis is mainly induced by interleukin (IL)-6^6^, IL-1β, and tumor necrosis factor (TNF)^7^.

**Figure 2:**
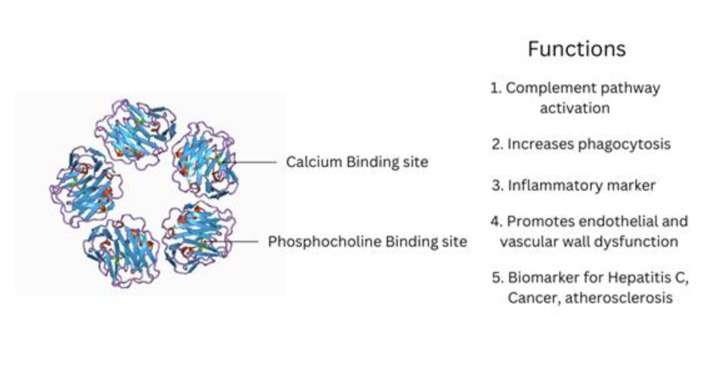
CRP Structure with phosphocholine and calcium binding sites and function

CRP binds to polysaccharides of many bacteria in the presence of calcium, which results in activation of the classic complement pathway and can promote phagocytosis and opsonization^8^(Figure 2).

**Figure 3:**
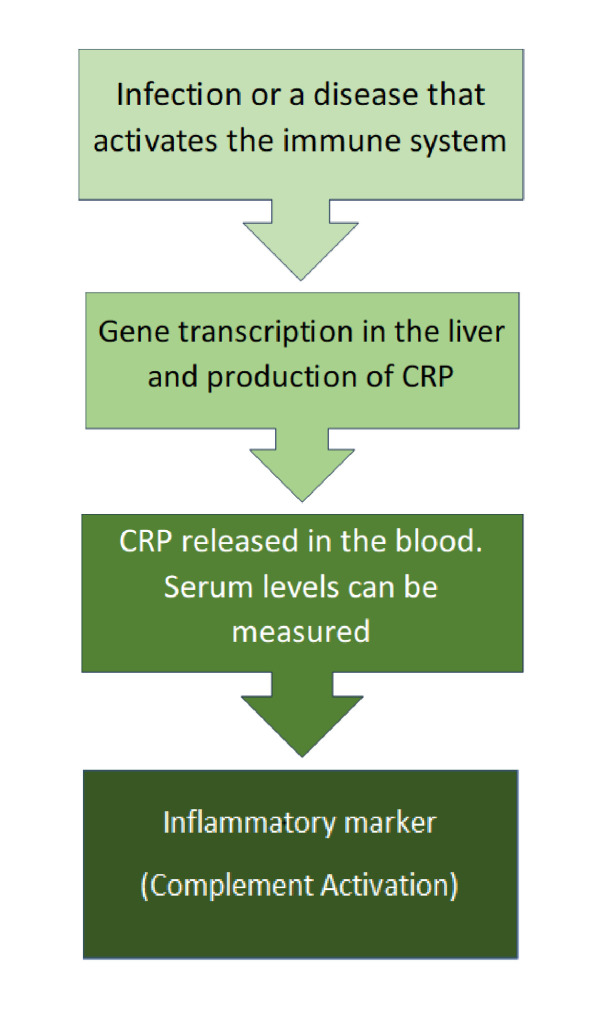
Steps leading to increased serum CRP levels

C-reactive protein (CRP) is an important component of the innate immune response. Its value deviates from the baseline by 25%^9^ and can increase 1000-fold following systemic infections, trauma, and malignancies^10^ (Figure 3). The average CRP level in Caucasians is 0.8 mg/l, however, many factors can change the baseline levels of CRP, including age, smoking status, and weight^11^ gene polymorphism, and a study found that–35-40% change can be hereditary^12^.

## 2. CRP in Disease Progression

### 2.1. CRP in cardiovascular disorders

**Figure 4:**
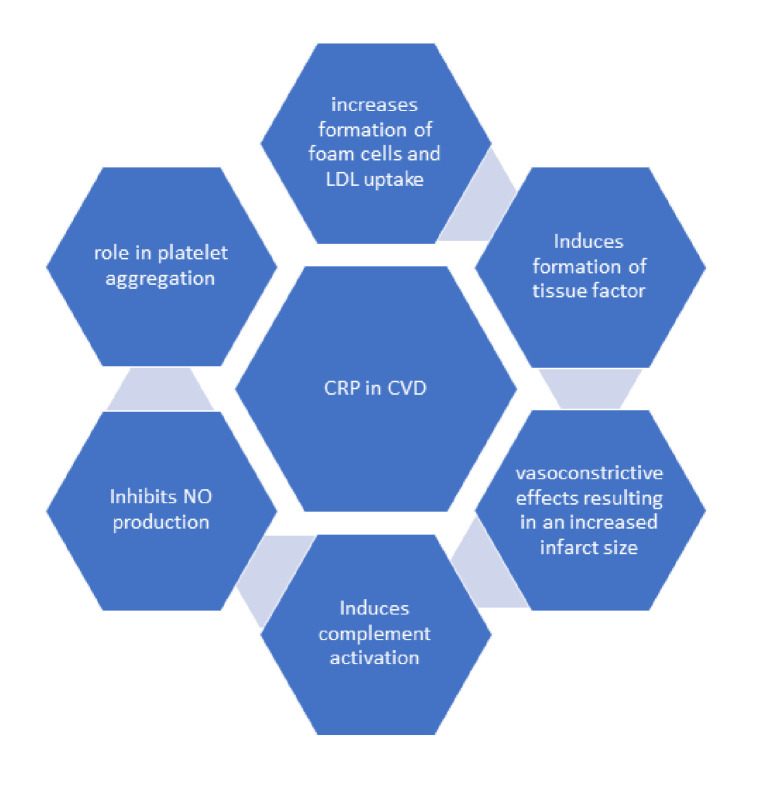
Representation of the role of CRP in cardiovascular disease

Many studies have discussed the importance of CRP levels in predicting the risk of cardiovascular disorders (CVD). Variation in plasma CRP levels, as an acute-phase protein, may show a retrospective view of an ischemic condition, trauma, or immune-mediated inflammation in the body, as CRP levels have been shown to deviate by approximately 25% from the original level after the onset of an inflammatory disorder^13^ (Figure 4). This has been employed to predict the trajectories and prognostic values of several cardiovascular diseases to provide adequate management and palliative care (Table 1).

**Table 1:** CRP Levels in Cardiovascular Pathologies

Cardiovascular Pathology	Manifestation of CRP levels
Atherosclerosis	Higher CRP levels accelerate atherosclerosis's primary development and progression^14^. CRP-induced activation of the immune system increases lipid accumulation and thus plaque formation^15^.CRP’s interaction with the endothelium mitigates nitric oxide formation causing plaque sensitivity with consequent vasoconstrictive effects resulting in an increased infarct size in myocardial infarction (MI)^14,16^.
Thrombogenicity	CRP at 10-100mg/L promotes platelet aggregation and indirectly activates tissue factor (TF) creating a hypercoagulable state^15^.
Major Adverse Cardiac Events (MACE)	Every 1mg/L of CRP was correlated with a 12% increased risk^17^.An elevated risk for cardiac mortality is seen after ST-elevation myocardial infarction with maximum danger at 2-3mg/L of CRP within a year of hospitalization^18^.Several studies still ascribe superiority to CRP to identify high-risk groups when talking about MACE, heart failure, and restenosis^17-20^.

However, when discussing CRP in the context of CVD progression, its classification as a mere downstream inflammatory marker due to the expression of IL-6, IL-1, and TNF-α has made it the focus of much scrutiny. Recently, a Mendelian randomization study conducted to clarify the role of CRP in predisposition to atherosclerosis was unable to find a direct correlation between genetic variants of CRP and coronary heart disease (CHD) risk. This relationship has been described as a reverse causation. This showed that genetically high CRP levels did not contribute as a CHD risk factor; rather, they acted as a secondary covariate associated with inflammatory responses to the disease^21^. Inconsistent trends between ethnic differences in CRP levels and the prevalence of major cardiovascular events^22^ have prompted clinicians to question their relevance.

Lastly, the ability of CRP to prognosticate recurrent MI episodes has recently been undercut by its high-half-life alternative, hs-CRP^23^.

### 2.2 CRP and Autoimmunity

The association of CRP with autoimmunity is highlighted when discussing the interaction of the protein with complement regulatory protein H to decrease the formation of membrane attack complexes^24^. This diminishes the chemo-attractant ability of C5a and contributes to the minimization of tissue damage at sites of inflammation. The contributory role of CRP in the development of autoimmunity is widely debated owing to reports of diminished numbers of pathogenic autoantibodies seen in those with prolonged low-dose exposure to CRP^25^; however, this remains speculative.

The most concrete evidence corroborating the above hypothesis is the role of CRP in the development of Systemic Lupus Erythematosus (SLE) (Table 2).

**Table 2:** The Role of CRP in SLE

Progression of SLE	Association with CRP
Immune Regulation	Increased CRP levels facilitate immune complex elimination^26^.
Gene polymorphism	Patients show a low basal CRP and insufficient CRP responses which are attributed to the presence of CRP-lowering gene polymorphism of rs105^27^.
Auto-antibody proliferation	Patients produce anti-CRP autoantibodies which primarily attack vulnerable epitopes after CRP dissociation^27^.

In juxtaposition, CRP levels manifest differently in rheumatoid arthritis (RA). RA, a chronic inflammatory condition mainly affecting the joints which is variably autoimmune in origin, has employed CRP as a marker of disease activity based on the core components of the 28-joint Disease Activity Score (DAS28). The involvement of CRP in RA progression is summarized in Table 3.

**Table 3:** The Destructive Role of CRP in Autoimmunity

Progression of RA	Association of CRP
Progressive Inflammation	Although a correlation has been seen between serum CRP levels and tissue inflammation in the knees, Patients have also shown increased levels of CRP in the synovial fluid. This could be due to augmented CRP signaling in fibroblast-like synoviocytes and could explain the progressive inflammation^28^.
Development of disease	Persistent elevations in CRP levels are seen in patients with RA (with levels >20mg/L) and a general decrease is seen due to medications^29^.
Correlation with other pathologies	Evidence shows radiological progression, increased risk of joint pathology, and reports of bone destruction due to an elevated baseline CRP^30,31^.
Prediction of complications	There is an increased cardiovascular risk, increased prevalence of metabolic syndrome, and diagnoses of comorbid diabetes mellitus have also been seen in RA patients with abnormal CRP levels^32-34^.There is a reported infiltration of the blood-brain barrier by elevations in mCRP secondary to autoimmune conditions like RA. This risks neuroinflammation and a possible progression to Alzheimer’s disease^35^ indicating the significance of CRP monitoring in patients with RA.

### 2.3. CRP in infection and sepsis

The manifestation of CRP levels in ongoing infectious processes, although is diagnostically important to differentiate between bacterial and viral infections, its role in determining the course of infection is also being extensively researched. CRP levels are used for risk stratification for COVID-19, Dengue Virus (DENV), and Human Immuno-deficiency Virus (HIV) infections (Table 4).

**Table 4:** Role of CRP in notable infections

Infective Agent	Role of CRP
COVID-19	As a product of cytokine induction, CRP has been classified as an indicator of cytokine storm^36^ which is one of the major reasons for mortality in COVID-19 patients. An increase in CRP levels in the initial phase of the infection has been implicated as an early and highly sensitive predictor for severe infection^37^A CRP value >41.8mg/L is indicative of the likelihood of developing severe symptoms^38^.CRP levels can predict the onset of comorbid cardiovascular conditions, cancer, and the probability of respiratory failure in patients^39-41^.
DENV	amplified CRP levels within the first 3 days of DENV infection are associated with unfavorable clinical outcomes, especially in children with a cut-off CRP value of 30.1mg/L^42^.Dengue patients have displayed higher levels of CRP than any other viral infection^43^.CRP levels signify susceptibility, fever clearance time, and risk of hospitalization in those suffering from the virus^42^.
HIV	Although HIV infection is not primarily an inflammatory condition, lower CRP levels have shown increased survival in patients.There is an inverse correlation between CD4 count and CRP and a direct correlation of CRP with HIV RNA. Higher levels have also indicated a faster progression to AIDS^44^.

Additionally, when discussing sepsis, the number of potential biomarkers is expansive owing to the intricacy of reactions perpetuating the event, but CRP is relatively widely used for assessing the risk of mortality, more specifically ICU mortality and organ failure^45^. According to Lobo et al., patients with CRP levels >10 mg/dl exhibited a significant increase in renal, respiratory, and coagulation failures, along with a definitively poorer prognosis in patients with sepsis than in those with CRP levels <1 mg/dL^46^. Serum analysis of CRP is coupled with leukocyte counts and albumin levels to obtain more accurate results.

### 2.4. CRP and cancer

**Figure 5:**
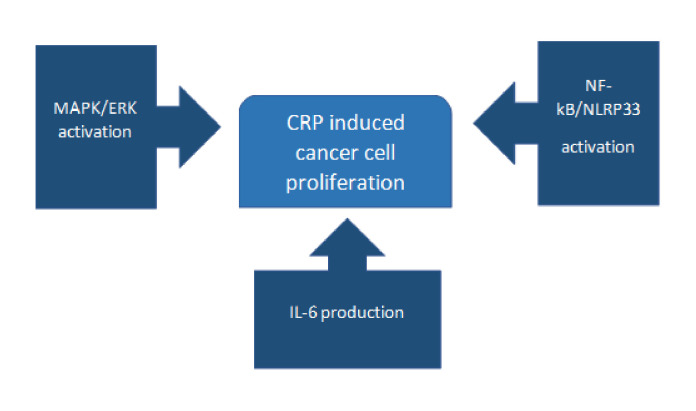
Representation of the role of CRP in cancer

Despite the lack of substantial evidence to support this, CRP has been linked to the question of cancer causation for many years. This is due to markedly elevated levels of CRP in the disease, known to cause increased cancer cell proliferation^47^, and the ability of the marker to accurately predict the risk of eventual cancer in previously healthy individuals^48^ (Figure 5). The literature has shown a significant association between increased serum CRP and the presence of breast, colorectal, ovarian, prostate, and lung cancers^49^. Other researchers have discussed the poor prognosis of melanoma, metastatic pancreatic cancer, and head and neck squamous cell carcinoma with increased CRP^50-52^. More recently, an increased risk of ovarian cancer was found with CRP > 10 mg/dl within 7 years of the assay^53^, and a high baseline CRP level (> 3 mg/L) implicated an 80% greater risk of early death from cancer^54^. CRP analysis in the context of colorectal cancer (CRC) has shown worse overall and cancer-free survival, with a low leukocyte-to-CRP ratio (LCR) and low CRP-albumin ratio, suggesting a definitive predictive relevance for stage II/III CRC and labeling LCR as the most sensitive biomarker for cancer progression^55^. Moreover, post-procedural CRP evaluation has shown promise in accurately predicting the peritoneal recurrence of CRC^56^.

## 3. **CRP in Diagnosis**

**Figure 6:**
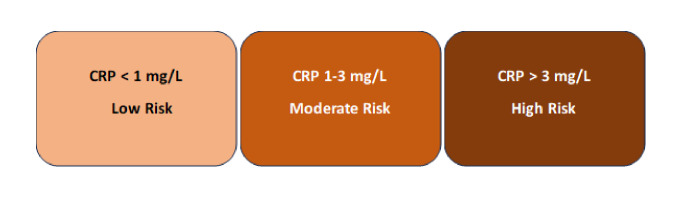
Individuals grouped into three categories based on CRP levels

As an acute-phase protein, inflammation-induced CRP release is the response of the body to tissue damage and injury, which refines the role of CRP as a measure of tissue damage decontextualized in the form of inflammation. The inability of the CRP response to differentiate between the cause and effect of the pathology and the gap in the understanding of its true function has left the significance of CRP as a diagnostic marker, largely to interpretation. However, the data suggest some unification in the results that has allowed its use despite ambiguity. Most data seemed to show the increase in CRP levels post pathology to be significant within hours to days from the onset of the insult varying from 10-100 folds within 6-72 hours of the initial damage, which is the foundation of its diagnostic relevance^4,57^ (Figure 6).

### 3.1. CRP in Infections and Sepsis

Although CRP lacks diagnostic specificity, it has been widely employed to differentiate between bacterial and viral infections and to classify bacterial infections based on severity. Viral infections were associated with a lower increase in CRP levels than bacterial infections. Several studies have classified this diagnostic strategy as ‘CRP velocity’ and how it positively correlated with bacterial febrile diseases^58^ Higher CRP concentrations are also seen in lower respiratory tract infections than viral infections. This may be demonstrated by the prevalence of CRP use in confirmatory tests for community-acquired pneumonia, with a reported cutoff value of > 20 mg/L showing the greatest accuracy^59^. In one study, CRP values > 25 mg/dL within a six-biomarker combination were able to diagnose severe bacterial infections in children with the highest sensitivity^60^. CRP has also been used as a diagnostic marker of acute appendicitis^61^.The use of CRP in diagnosing acute neonatal sepsis has shown results with a sensitivity of 76.92%^62^, whereas in adults, CRP was able to diagnose and differentiate between sepsis with and without bacteremia, especially when used in conjunction with procalcitonin^63^. Many developments in the rapid immunoassay of CRP for the diagnosis of neonatal sepsis are underway, which can only emphasize the diagnostic precision that it allows when discussing sepsis. Array-based electrochemical magneto-immunosensors have demonstrated promising results^64^. Despite this, the place of the CRP assay in emergencies is currently under debate^65^.

### 3.2. CRP in Osteomyelitis

Osteomyelitis is one of the most prevalent complications of diabetes. Its specific and extreme implications and treatment options make accurate and timely diagnosis imperative. CRP level and erythrocyte sedimentation rate (ESR) were used collectively for this purpose. While ESR serves as a more sensitive option, CRP has been reported to have the ability to differentiate between osteomyelitis and soft tissue infection, with a cutoff value of >7.9 mg/dL^66^. A recent meta-analysis highlighted CRP as 68.5% sensitive and 70.6% specific for diagnosing diabetic foot osteomyelitis^67^.

### 3.3. CRP in Inflammatory Bowel Disease

Evidence has brought CRP to the spotlight when discussing the diagnosis of inflammatory bowel disease (IBD), providing an appropriate workup of activity in the acute phase^68^. The positive correlation between CRP and microorganisms in the blood providing the pro-inflammatory stimulus and the strong association of CRP albumin ratio with disease progression conclusively point to its utility in IBD diagnosis and monitoring of treatment^69^. CRP has been reported to have 78.9% sensitivity and 85.7% specificity when measured in the context of IBD, but the marker still exhibits poor clinical use owing to the unreliability of the circumstances inducing its release^70^.

## 4. CRP in disease management

**Figure 7:**
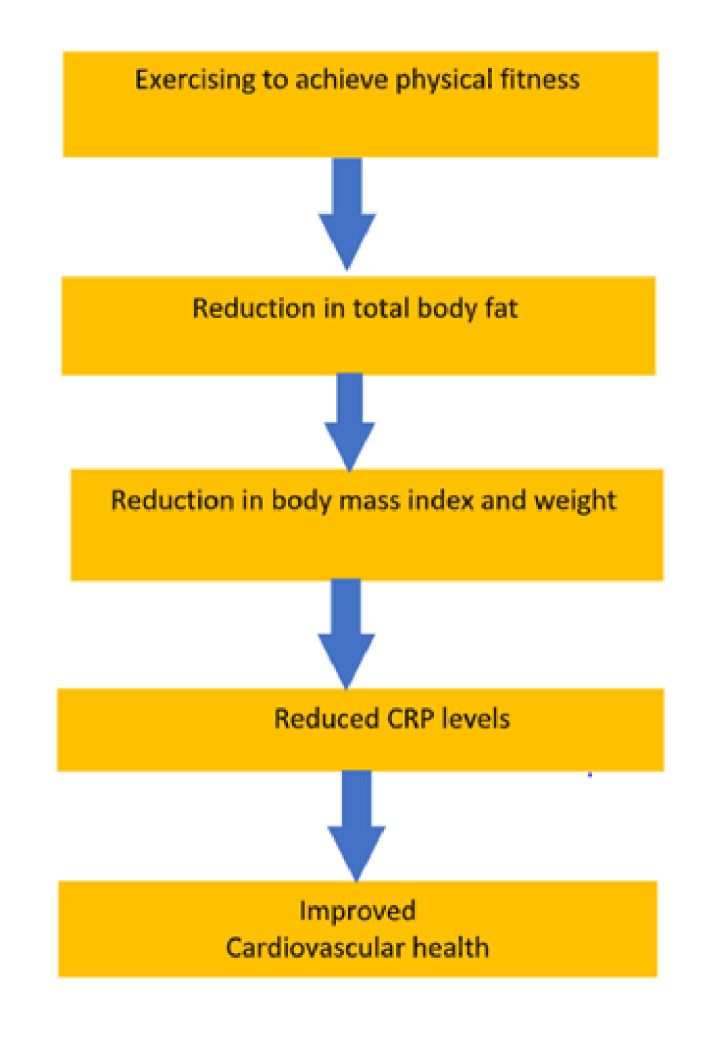
Implication of CRP in cardiovascular fitness^72^

The risk of atherosclerosis superimposed with thrombotic events is caused by two principal factors: hyperlipidaemia and inflammation. However, in a recent study published in collaboration with international randomized trials (PROMINENT, REDUCE-IT, and STRENGTH) in association with the American College of Cardiology, it was concluded that hs-CRP (high sensitivity-CRP) levels, which measure inflammation, have a greater predictive effect on atherosclerotic cardiovascular disease and mortality than LDL-cholesterol levels in patients already receiving cholesterol-lowering statins^71^. While lipid-lowering drugs to combat hypercholesterolemia are indispensable in treating and reducing cardiovascular disease risk, evidence from trials suggests that the fundamental role of the inflammatory marker CRP should be acknowledged and employed to assess cardiovascular risk, in addition to the residual risk in patients with CVD (Figure 7). The primary future implication is that clinicians should consider both therapeutic strategies of lowering blood cholesterol levels and decreasing inflammation as having a mutually beneficial relationship in improving patient health, rather than the two strategies being used independently for treatment.

For a comprehensive global evaluation of the risk of heart disease and its related comorbidities, measuring CRP level is a reliable method employed by clinicians. Individuals were grouped into low-, moderate-, and high-risk categories based on CRP values of less than 1, 1-3, and greater than 3 mg/L, respectively^73^. CRP is used as an individual marker for atherogenesis, myocardial infarction, stroke, and cardiac arrest due to coronary heart and peripheral arterial diseases. High-sensitivity CRP is used to assess cardiovascular risk, as older tests are more suitable for detecting advanced pathological inflammatory states and are less sensitive to risk discernment. A CRP test along with a lipid panel for cholesterol measurement is necessary for patients belonging to the high CRP/low LDL category with a higher probability of developing CVD than for individuals falling in the low CRP/high LDL bracket, who would have been overlooked based on a cholesterol test alone^74^.

A CARDIA study conducted on 4405 individuals between the ages of 18-30 years proposed that management and reduction of CRP levels were positively correlated with favorable cardiac and circulatory health during the progression from adolescence to adulthood^75^.

A study showed that the best treatment for Crohn’s would be accomplished by using both CRP and Fecal Calprotectin (FCP) together^76^. The importance of assessing diseases such as Crohn’s disease using inflammatory markers such as CRP is highlighted by the fact that infiltrative techniques that are prone to imprecision are more commonly employed in clinical practice. The usual practice to measure the extent of infection in inflammatory bowel disease is to perform a colonoscopy that requires extensive preparation, instrumentation, and risks of perforation as well as allergic reactions, among many others. Although colonoscopy is the mainstay for the diagnosis of Crohn’s disease, CRP as a biomarker for Crohn’s along with Fecal Calprotectin (FCP) has been shown to reduce the need for colonoscopy and its associated complications. However, increased levels of both biomarkers in the setting of reduced symptoms of disease requires confirmation with endoscopy, as does monitoring for increased severity and dysplastic changes in the epithelium^77^​​. This emphasizes the importance of relying on biological markers for disease management before resorting to instrumentation.

A salient contribution of CRP in tracking symptom improvement in patients with Crohn’s disease receiving infliximab was observed in a previous study. A positive correlation was found between high baseline CRP levels greater than 15 mg/dl and continuance of symptoms without improvement, highlighting the ineffectiveness of infliximab therapy and prolongation of disease symptoms with higher CRP levels before treatment^78^.

Serious complications of colorectal surgery can be avoided if CRP levels are monitored during surgery, as the incidence of anastomotic leakage is correlated with increased CRP levels^79^.

The C-reactive protein to albumin ratio (CAR) has also been useful in estimating the probability of post-liver transplant complications and death^80^.

Periodic and regular CRP level measurement is a potent and advantageous technique for gauging the success of therapeutic strategies for inflammatory bowel disease (IBD). CRP surpasses Fecal Calprotectin (FCP) in its dominance over the recognition and management of functional gastrointestinal disorders (FGIDs), such as IBS ^81^. A range of studies suggest that decreased CRP levels are indicative of the course of improvement of a disease condition, prediction of recovery, better future health, and an ameliorated patient response to treatment^81^ ^82^. According to a study that monitored patients with IBD, CRP levels were more specific for Crohn’s than for ulcerative colitis. CRP levels also predict the likelihood of surgery in patients with both ulcerative colitis and Crohn’s disease^83^.

Modulation of CRP levels and vigilant assessment can also prevent autoimmune disorders such as rheumatoid arthritis, where elevated CRP levels are suggestive of aggravation of symptoms. CRP is an essential element of many RA-related scales, indices, and criteria that provides a comprehensive understanding of disease progression and treatment^84^.

A systematic review of biomarkers associated with RA concluded that a substandard response to conventional synthetic disease-modifying antirheumatic drugs (csDMARDs) for RA was indicated with CRP levels >7.1 mg/L^85^.

**Figure 8:**
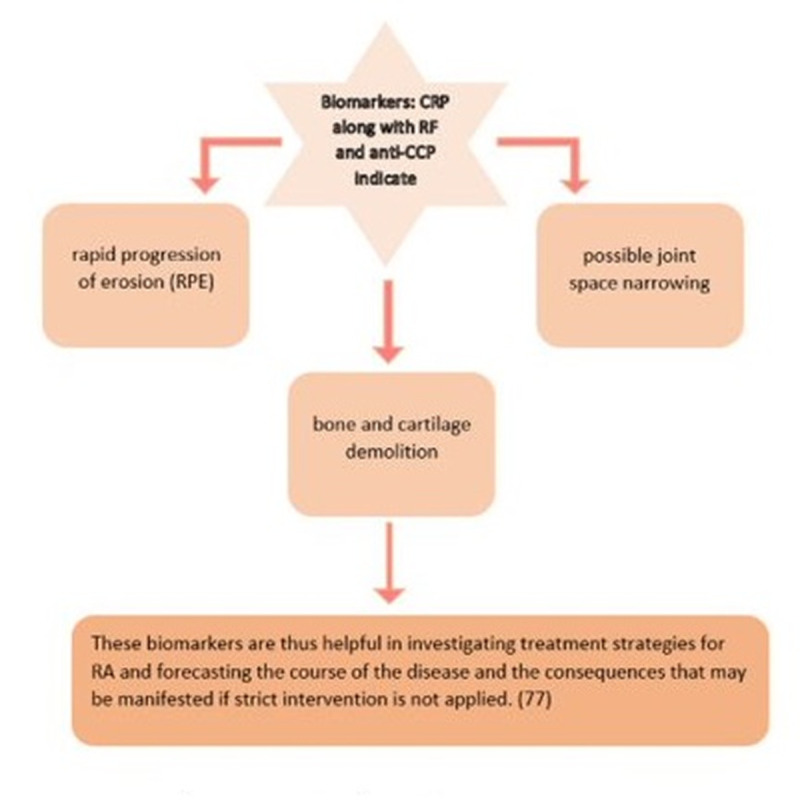
CRP and management of Rheumatoid Arthritis

CRP, rheumatoid factor (RF), and anti-CRP antibodies help investigate treatment strategies for rheumatoid arthritis^85^ indicated by the following diagram (Figure 8).

Studies attributing depression to pervasive inflammation have implied that high concentrations of highly sensitive hs-CRP may be detected in blood and cerebrospinal fluid^86^.

In a study to detect the presence of secondary infection in COVID-19 patients using CRP and PCT, it was suggested that a rise or fall in CRP levels can predict the presence or absence of hospital-acquired infection in patients with COVID-19 and may necessitate antibiotic therapy^87^.

CRP point-of-care testing is recognized as an important test used in outpatient departments to reduce reliance on antibiotics^88^. Patients with a lower respiratory tract infection and prominent symptoms of fever with high CRP levels were directed towards antibiotics in a CRP Point-of Care Testing (POCT)^89^.

The C-reactive protein-to-albumin ratio (CAR) was recognized in a recent study as a marker of respiratory failure in Guillain Barre syndrome, with CAR>0.21 having a positive correlation and CAR>0.19, coinciding with an increased risk of aggressive disease with a low prospect of recuperation^90^.

If effectively controlled, the CAR ratio can also prevent respiratory complications in P. falciparum malaria.

Increased CRP to Albumin Ratio in adults has implicated worse prognosis and respiratory complications due to imported falciparum malaria^91^.

**Figure 9:**
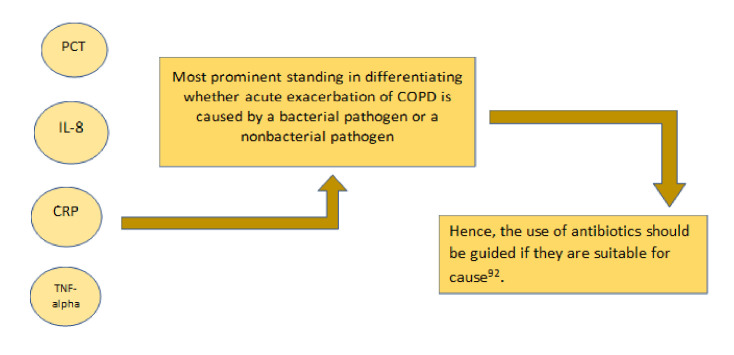
CRP and management of COPD

A systematic review and meta-analysis compared CRP, procalcitonin (PCT), IL-8, and TNF-α in chronic obstructive pulmonary disease (COPD) with the following results^92^(Figure 9).

A study that measured CRP levels in the saliva and serum of children with acute respiratory illness suggested a positive correlation between salivary and serum CRP levels, suggesting that salivary CRP accurately indicates high serum CRP levels, thereby virtually minimizing the need for phlebotomy in children^93^.

As rising CRP levels have been positively implicated in a plethora of inflammatory diseases, a sensible assumption would be to reduce these levels to restrict the progression of these diseases and possibly help provide more proficient treatment. Various studies have shown that reducing the hepatic synthesis of CRP by directly inhibiting CRP mRNA translation successfully lowered CRP levels using Anti Streptolysin-O titers (ASO) in humans and experimental rats^94^.

Another approach utilizing 1,6-bis(phosphocholine)-hexane reduced the size of infarcts and exhibited a cardioprotective role by suppressing the exacerbation of the negative effects in experimental mice in which myocardial infarction was stimulated. This was achieved by preventing the binding of CRP to its ligands, enhancing its excretion, and reducing complement-mediated inflammatory effects^95^. However, CRP also exhibits the potential to aggregate with 1,6-bis (phosphocholine)-hexane molecules and accumulate in the vessels, causing detrimental effects and possibly activating further inflammatory side effects. Immunosuppression is a more threatening clinical consideration when targeting CRP for disease management using this compound. CRP apheresis is an effective extracorporeal therapeutic advancement that can be employed to directly direct its effects on CRP without interfering with the levels of other markers of inflammation^96^. This technique proved successful in lowering CRP levels in patients with myocardial infarction but showed nonspecific results in patients with COVID-19^97^.

To test whether CRP lowering can have beneficial effects in the management of acute kidney injury and in patients receiving kidney transplants, a study using CRP transgenic mice compared to wild-type mice explored the effects of inducing ischemia-reperfusion injury in these mice. The results indicated that targeting CRP for kidney injury can be a valuable intervention, since it was shown to exacerbate tubular damage and promote its transition from acute to chronic kidney disease^98^. Transgenic mice before ischemia-reperfusion injury did not show grossly elevated CRP levels and thus did not display significant clinical manifestations of Acute Kidney Injury either^99^.

The link between inflammation and malignancy has been corroborated and reinforced by various studies. Elevated CRP levels are strong indicators of poor prognosis in cancer treatment. A study on patients with oesophageal cancer emphasized the prognostic value of serum CRP in assessing patient response and survival^100^. A study observing the quality of life of patients post-treatment for endometrial cancer suggested that CRP measurement, along with other markers such as GPS and CAR pre- and postoperatively, has a notable prognostic significance in judging disease-related mortality, recurrence, management, and response to treatment^101^.

Shinohara et al.’s study on lung cancer also showed improved patient survival in the group of patients with CRP levels <5 mg/L after surgery^102^.

Hence, CRP assessment has helped in increasing success in cancer survival and mitigation of worsening exacerbation or recrudescence of the disease.

## 5. Limitations and future discussion

The indisputable potential of CRP as a marker for diagnosis, disease progression, prognostic indicators, and follow-up of outcomes to treatment regimens has been authenticated by numerous studies on the pathogenic mechanisms and prevention of various diseases.

The novel role of CRP in appraising the success of medical interventions for treating IBD has been demonstrated; however, it is less reliable in patients with milder diseases who may not have significant elevations in the diagnosis of mucosal healing^103^.Leucine-rich alpha-2 glycoprotein is regarded as a more important biomarker than CRP for tissue damage and inflammation in Crohn’s disease^104^.

Multiple studies have reported that CRP level alone is not sufficient to predict morbidity or mortality in disease states, and CAR provides a more accurate prediction^105^. Nonetheless, the specificity of CAR is compromised because CRP and albumin levels are independently affected by different factors.

A study has also proposed that the invasive method of assessing CRP levels for cardiovascular disease from a blood sample can be replaced with the Bi-Digital O-Ring Test Resonance Phenomenon using L-homocysteine, especially for circumstances in which CRP levels are not indicative of any underlying pathology^106^.

High CRP levels are found in certain neurodegenerative diseases such as Alzheimer’s disease, Parkinson’s disease, and age-related macular degeneration^107^. A comprehensive understanding of how CRP levels are associated with the development and progression of these diseases is not completely understood or ascertained in comparison with its clear association with more prevalent disorders, as discussed above. Further exploration is imperative to establish the importance of CRP as a prognosticator for effective management of these diseases.

The role of CRP in the diagnosis of complicated disease processes and investigating effective treatment modalities for them cannot be negated; nevertheless, it is imperative to mention that it is a non-specific marker of inflammation, which may either be the causative factor underlying the pathogenesis of a disease, simply associated with it, or come forth as one of its associated complications. Hence, sole reliance on the measurement of this inflammatory marker to understand disease causes, progression, and treatment response is often neglected as more specific markers for diseases have been identified and compared in their effectiveness with CRP. Having established that the most effective clinical investigations regarding diseases employ multiple testing parameters assessing CRP along with disease-specific markers to comprehensively probe the complexities that underlie the diseases, and that relying on either disease marker alone would not yield.

## 6. Conclusion CRP

In conclusion, this review aimed to provide a comprehensive understanding of the C-reactive protein (CRP) role as a biomarker for disease progression, diagnosis, and management. Studies have indicated that changes in CRP levels can reflect the severity of inflammation and predict the risk of developing chronic inflammatory disease. The significance of CRP as a diagnostic tool is enhanced by understanding its association with the development of diabetes, cardiovascular disease (CVD), and autoimmune diseases. CRP levels are predictive of future outcomes in various populations including healthy individuals and high-risk patients. Despite the constraints in interpreting the CRP results, including the necessity for multiple tests and potential interfering variables, its practical value cannot be disregarded. In general, understanding the effects of CRP levels on disease progression, diagnosis, and management can result in enhanced patient care and outcomes.

## Bullet points

- *Erythematosus and pathogenic role in the development of Rheumatoid Arthritis.*

- *CRP levels help investigate treatment strategies for rheumatoid arthritis. Substandard response to treatment is seen with CRP levels >7.1 mg/L.*
